# Transitioning CAR‐T therapy for multiple myeloma to the outpatient setting: First clinical insights with cilta‐cel in Germany

**DOI:** 10.1002/hem3.70417

**Published:** 2026-07-01

**Authors:** Christof Scheid, Philipp Gödel, Christopher Krone, Elisa Carpinteiro, Michael Hallek, Udo Holtick, Tim Richardson

**Affiliations:** ^1^ Department I of Internal Medicine, Faculty of Medicine and University Hospital Cologne University of Cologne Cologne Germany

B‐cell maturation antigen (BCMA)–directed immunotherapies have fundamentally changed the treatment landscape of multiple myeloma (MM).^1^, ^2^ Ciltacabtagene autoleucel (cilta‐cel) has demonstrated high response rates and durable remissions in patients with relapsed or refractory disease and is increasingly used in routine clinical practice.^3^, ^4^ Alongside chimeric antigen receptor T‐cell (CAR‐T) therapy, T‐cell–redirecting bispecific antibodies (BsAbs) are becoming widely available, further expanding immune‐based treatment strategies in MM and rapidly moving into earlier lines of therapy.^5^ As a result, the number of patients eligible for immune effector cell therapies is steadily increasing.

Despite this evolution, CAR‐T therapy is commonly delivered with prolonged inpatient monitoring, largely driven by concerns regarding acute toxicities such as cytokine release syndrome (CRS) and immune effector cell–associated neurotoxicity syndrome (ICANS), as well as reimbursement structures linked to hospitalization in some healthcare systems.^6^, ^7^ In Germany, inpatient treatment has traditionally been required for CAR‐T reimbursement, resulting in routine hospital stays of up to several weeks even in clinically stable patients. Against the background of increasing treatment volumes and growing pressure on healthcare resources, strategies that safely reduce hospital utilization are urgently needed.^8^ In this context, some US centers have implemented early‐discharge or hospital‐based outpatient management programs as reported, for example, by the Mayo Clinic in Rochester,^9^, ^10^ whereas such approaches have remained uncommon in Germany and most European healthcare systems.

At our tertiary care center, cilta‐cel has been administered to approximately 150 in‐label MM patients with prolonged inpatient monitoring in line with established practice. With growing institutional experience in CAR‐T therapy and toxicity management, we implemented a structured fully outpatient treatment pathway for selected patients. The aim was to avoid routine hospitalization while maintaining a low threshold for inpatient admission whenever clinically indicated.

Eligibility for outpatient treatment was determined by predefined clinical and logistical criteria, including reliable adherence, Eastern Cooperative Oncology Group (ECOG) performance status of 0–1, adequate organ function assessed by echocardiography and pulmonary function testing, absence of relevant cardiovascular or neurologic comorbidities, low tumor burden as assessed by paraprotein levels and imaging or adequate response to bridging therapy, travel time of less than 60 min to the treatment center, and availability of a dedicated caregiver living in the same household. All patients provided informed consent for outpatient management. Low tumor burden was defined in a composite, clinically pragmatic manner: patients were required to have no evidence of extramedullary disease on imaging (positron emission tomography–computed tomography [PET–CT]), no features of high disease activity (such as hypercalcemia or myeloma‐related renal impairment), and either an adequate response to bridging therapy or stable, low‐volume disease. Response to bridging was a central criterion: 9 of 12 patients (75%) had achieved at least a partial response, including 4 patients (33%) who reached a very good partial response or better prior to infusion. The other three patients had a stable/progressive disease with low tumor burden (Table 1).

**Table 1:** Baseline patient characteristics, outcomes, and used therapy for outpatient ciltacabtagene autoleucel (cilta‐cel) treatment: bridging therapy regimens are reported as abbreviations: DKd, daratumumab/carfilzomib/dexamethasone; EPd, elotuzumab/pomalidomide/dexamethasone; DPd, daratumumab/pomalidomide/dexamethasone; KD, carfilzomib/dexamethasone; PCd, pomalidomide/cyclophosphamide/dexamethasone.

Characteristic	Value (*n* = 12)
Age at infusion, years	61 (47–73)
Sex, *n*	Male 10; female 2
ECOG performance status at CAR‐T, *n* (%)	0: 9 (75%); 1: 3 (25%)
ISS stage at diagnosis, *n* (%)	I: 3 (25%); II: 4 (33.3%); III: 5 (41.7%)
High‐risk cytogenetics, *n* (%)	6 (50%)
Median number of prior lines of therapy	2 (1–9)
Triple‐ or penta‐refractory disease, *n* (%)	8 (66.7%)
Prior BCMA‐directed therapy, *n* (%)	2 (18%)
Bridging therapy administered, *n* (%)	11 (91.7%)
Bridging therapy regimen, *n* (%)	DKd: 5; EPd: 2; DPd: 1; KD: 1; PCd: 1; none: 1
Response to bridging therapy, *n* (%)	≥PR: 9 (75%); SD/PD: 3 (25%)
Vein‐to‐vein time, days	88 (55–123)
Baseline creatinine at CAR‐T, mg/dL	0.86 (0.59–1.19)
*Outcomes*	
CRS Grade 1, *n* (%)	8 (66.7%)
CRS Grade 2, *n* (%)	1 (8.3%)
CRS Grades 3 + 4, *n* (%)	0
ICANS, *n* (%)	0
Hospitalization within 30 days, *n* (%)	4 (33.3%)
Length of inpatient stay, days	3 (1–5); total 12 days
Best overall response, *n* (%)	CR 11 (91.7%); SD 1 (8.3%)
*Therapy*	
Dexamethasone 10 mg, *n* (%)	9 (75%)
Median days of dexamethasone	1 (1–2)
Tocilizumab, *n* (%)	4 (33.3%)
Median days of tocilizumab	1 (1–2)
Antibiotic therapy, oral, outpatient	1 (8.3%)
Antibiotic therapy, intravenous, inpatient	2 (16.7%)

Abbreviations: BCMA, B‐cell maturation antigen; CAR‐T, chimeric antigen receptor T cell; CR, complete remission; CRS, cytokine release syndrome; ECOG, Eastern Cooperative Oncology Group; ICANS, immune effector cell–associated neurotoxicity syndrome; ISS, International Staging System; PD, progressive disease; PR, partial remission; SD, stable disease.

Lymphodepleting chemotherapy with fludarabine and cyclophosphamide was administered in the outpatient setting according to standard dosing schedules. Cilta‐cel infusion was performed at the treatment center on Day 0, followed by a 6‐h observation period. Thereafter, patients were monitored through a structured outpatient surveillance program consisting of in‐person visits three times per week including clinical examination and laboratory testing, daily telephone contact by trained medical staff, and patient‐performed monitoring of vital signs and Immune Effector Cell–Associated Encephalopathy (ICE) scores at home. We defined outpatient treatment as initial management without planned inpatient monitoring, with predefined criteria for immediate hospital admission if clinically indicated.

Continuous 24/7 access to CAR‐T–experienced physicians was ensured, with immediate availability of the emergency department, intensive care facilities, and anti–IL‐6 therapy. Patients were provided with oral standby antibiotics for neutropenic fever (according to MASCC score) and dexamethasone for use only after medical consultation.^11^ Fever or other signs of toxicity prompted immediate evaluation, with a deliberately low threshold for inpatient admission either via the emergency department or electively from outpatient follow‐up. Clinical situations prompting hospitalization were: continuous high fever despite dexamethasone, CRS ≥ Grade 2, any signs of ICANS, an absolute lymphocyte count peak ≥ 3 × 10^9^/L, or at patient's request. An ALC peak ≥ 3 × 10^9^/L was included as a predefined trigger based on evidence linking pronounced post‐infusion lymphocytosis after cilta‐cel to higher rates of delayed neurotoxicity.^12^ CRS and ICANS were graded according to ASTCT consensus criteria.

Between August 2023 and June 2025, 12 selected patients with relapsed or refractory MM were treated with cilta‐cel in an outpatient setting and retrospectively analyzed. Baseline patient characteristics are summarized in Table 1 and Figure 1. Median age was 61 years (range 47–73), median ECOG performance status was 0 (range 0–1), and patients had received a median of two prior lines of therapy before apheresis (range 1–9). Median vein‐to‐vein time was 88 days (range 55–123). All patients fulfilled predefined eligibility criteria and completed lymphodepleting chemotherapy and CAR‐T infusion without upfront hospitalization. During the same period, approximately 110 patients received cilta‐cel at our center under standard inpatient monitoring, which represented the default pathway for all patients; the outpatient program was not universally offered but selectively proposed to patients meeting predefined clinical and logistical eligibility criteria. An additional approximately 15 patients were offered the outpatient pathway but preferred inpatient monitoring, either fully or with at least inpatient CRS surveillance beginning from Day +4 after CAR‐T infusion; this preference was fully respected. The most common reasons for patients not entering the outpatient pathway were patient preference for inpatient monitoring, ECOG performance status greater than 1, high tumor burden or insufficient response to bridging therapy, relevant cardiovascular or neurologic comorbidities, travel time exceeding 60 min, or absence of a dedicated in‐home caregiver.

**Figure 1:**
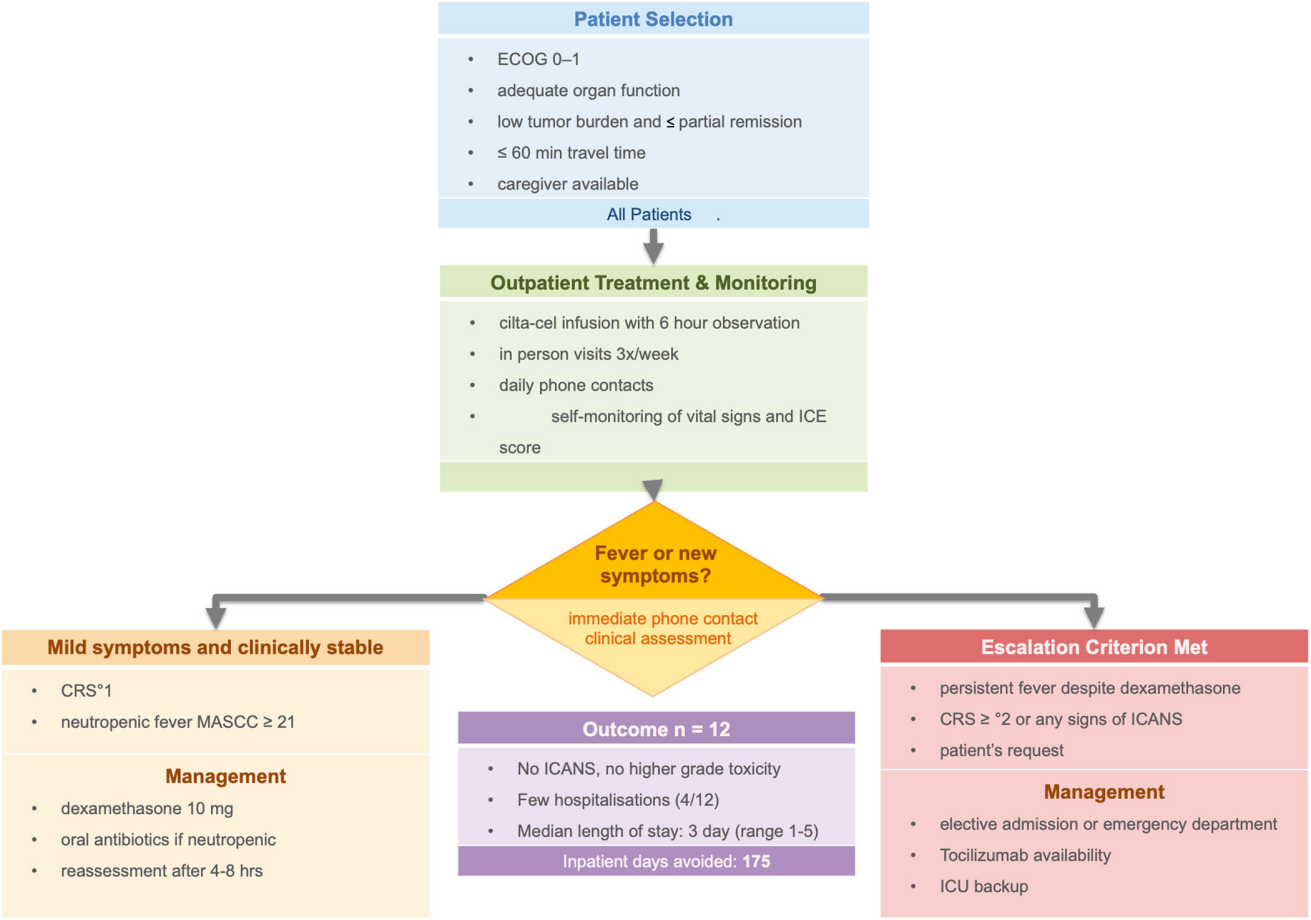
**Patient selection and decision tree, including relevant clinical outcomes for the 12 reported patients.** Fever management followed a predefined stepwise algorithm: telephone assessment, oral dexamethasone at home, reassessment at 4–8 h, and emergency department referral for persistent or escalating toxicity. Tocilizumab was reserved exclusively for the inpatient setting. cilta‐cel, ciltacabtagene autoleucel; CRS, cytokine release syndrome; ECOG, Eastern Cooperative Oncology Group; ICANS, immune effector cell–associated neurotoxicity syndrome; ICE, Immune Effector Cell–Associated Encephalopathy; ICU, intensive care unit.

Acute toxicities were manageable within the outpatient framework. CRS occurred in nine patients and was Grade 1 in eight cases and Grade 2 in one case. No patient developed ICANS. Fever was the most common presenting symptom during early follow‐up. All patients who developed fever at home contacted the treating physicians immediately by telephone. After clinical assessment and confirmation of clinical stability, dexamethasone 10 mg was initiated, followed by structured reassessment after 4–8 h. One neutropenic patient initiated oral antibiotic therapy at home after medical consultation. If the fever resolved and the patient remained clinically stable after reassessment, outpatient monitoring was continued. If fever persisted despite dexamethasone, if hemodynamic instability or clinical deterioration occurred, or if CRS Grade 2 or higher was suspected, patients were directed immediately to the emergency department. Tocilizumab was reserved exclusively for the inpatient setting and was not administered in the outpatient context; all four patients who required hospitalization received tocilizumab in the emergency department, which had been informed in advance, with rapid symptom resolution after a single dose in each case.

Within the first 30 days after infusion, four patients underwent short‐term inpatient hospitalization (33.3%). Importantly, inpatient admission was anticipated within the outpatient concept and followed predefined safety criteria. Two patients presented to the emergency department due to fever and were admitted at their own request, while two patients were electively admitted following a second consecutive day of fever during scheduled outpatient follow‐up. Two patients received intravenous antibiotic therapy after admission. Median length of inpatient stay was 3 days (range 1–5), accounting for a total of 12 inpatient days across all hospitalized patients. All hospitalizations occurred within the early post‐infusion period consistent with expected CRS timing. In comparison, the median inpatient stay in our institutional cohort of cilta‐cel‐treated patients was 16 days, resulting in an estimated 168–240 inpatient days for a comparable cohort of 12 patients translating in a reduction of more than 90% in inpatient bed utilization.

Reasons for hospitalization were Grade 2 CRS in one patient, persistent fever in one patient, and persistent fever with neutropenia in two patients. Tocilizumab was administered to all hospitalized patients, resulting in rapid resolution of symptoms after a single dose. Intravenous antibiotics were given to two hospitalized patients.

After a median follow‐up of 9.9 months (range 3.1–23.9 months), no patient had deceased or required intensive care admission, and non‐relapse mortality was 0%. The best overall response was a complete response in 11 patients and stable disease in one patient with prior CAR‐T therapy.

In this single‐center experience, we demonstrate that administration of cilta‐cel in an outpatient setting is feasible and safe in selected patients with MM when embedded in a highly structured monitoring and safety framework. To our knowledge, this is the first European report describing a structured outpatient program for cilta‐cel in MM.

While outpatient management of CAR‐T treatment is widely accepted in other healthcare systems in particular in the United States, in Europe, it is generally performed in the inpatient setting.^13^ However, several reasons make cilta‐cel a particularly suitable candidate for outpatient application: CRS is typically Grade 1 or 2 and severe CRS as well as ICANS is rare.^3^, ^14^ Risk factors for acute toxicities in patients undergoing cilta‐cel therapy are increasingly well defined and typically represent high tumor burden and poor performance status as well as baseline inflammation.^15^ In addition, the median time of CRS occurrence is Day 7 after infusion, often coinciding with recovery from neutropenia induced by lymphodepleting chemotherapy. Thus, there is much less risk to confound CRS with neutropenic fever with the need of empirical antibiotics and the risk of sepsis. Therefore, a risk‐adapted approach should allow for outpatient care while preparing a safety net for the event of severe complications.

A central component of our strategy was stringent patient selection combined with proactive outpatient surveillance and a deliberately low threshold for inpatient admission. Fever, the most frequent early toxicity, was managed through immediate physician contact, structured use of dexamethasone (typically 1–3 doses), and close reassessment, allowing the majority of cases to be handled without hospitalization. When inpatient care was required, admissions were short and driven by predefined safety considerations rather than uncontrolled complications. No patient required intensive care support, and no early mortality was observed.

Beyond clinical feasibility, outpatient administration of cilta‐cel has relevant implications for healthcare resource utilization.^16^ By avoiding routine inpatient monitoring periods of 14–20 days per patient, substantial numbers of hospital bed days were spared without compromising patient safety.

While limited by its single‐center design and small patient number, our report provides a pragmatic blueprint for outpatient cilta‐cel administration. Our program builds on extensive prior institutional experience with approximately 150 in‐label cilta‐cel‐treated patients, which may be a prerequisite for safe outpatient implementation. The feasibility observed in our cohort reflects stringent selection criteria and substantial prior institutional experience with CAR‐T therapy. These results should therefore not be generalized to unselected patient populations or centers without established CAR‐T infrastructure. Although no patient required intensive care support and no early mortality was observed, the small sample size precludes firm conclusions regarding rare but potentially severe toxicities. Furthermore, we acknowledge that the home monitoring setting inherently differs from continuous inpatient surveillance: transient Grade 2 CRS features, such as brief oxygen desaturations or self‐limiting hypotensive episodes occurring between monitoring intervals, cannot be fully excluded and may have been underdetected. The low CRS severity observed is, however, biologically consistent with the stringent patient selection applied and with published real‐world cilta‐cel data in lower risk populations.

Our data support consideration of outpatient treatment models for selected patients in experienced CAR‐T centers with structured monitoring programs and predefined admission pathways. Broader adoption of such models may improve patient experience, reduce nosocomial infections, and alleviate pressure on inpatient resources as CAR‐T therapy continues to expand. We are currently evaluating a similar outpatient strategy for BsAb‐treated patients.

## AUTHOR CONTRIBUTIONS

**Christof Scheid**: Conceptualization; supervision. **Philipp Gödel**: Data curation; writing—original draft; writing—review and editing; visualization. **Christopher Krone**: Data curation; formal analysis. **Elisa Carpinteiro**: Data curation; visualization; validation; writing—review and editing. **Michael Hallek**: Supervision. **Udo Holtick**: Writing—review and editing; methodology; resources. **Tim Richardson**: Writing—original draft; data curation; writing—review and editing; investigation; conceptualization; project administration.

## CONFLICT OF INTEREST STATEMENT

C. Scheid: Advisory Board for Sanofi, Johnson & Johnson, Kite, and Astrazeneca. P. Gödel: no conflict of interest to declare. C. Krone: no conflict of interest to declare. E. Carpinteiro: no conflict of interest to declare. M. Hallek: no conflict of interest to declare. U. Holtick: Advisory Board for Johnson & Johnson, Oncopeptides, Sanofi, and Jazz. T. Richardson: Advisory Board for Sanofi, Takeda, Oncopeptides, Kite, and Johnson & Johnson.

## ETHICS STATEMENT

This retrospective analysis was conducted in accordance with the Declaration of Helsinki. All patients provided written informed consent for outpatient management and for the use of their anonymized clinical data for research purposes. Ethical approval was obtained from the Ethics Committee of the University of Cologne, reference 24‐1201‐retro.

## FUNDING

This research received no specific grant from any funding agency in the public, commercial, or not‐for‐profit sectors. Open Access funding enabled and organized by Projekt DEAL.

## Data Availability

The data that support the findings of this study are available on request from the corresponding author. The data are not publicly available due to privacy or ethical restrictions.

## References

[1] Swan D , Madduri D , Hocking J . CAR‐T cell therapy in multiple myeloma: current status and future challenges. Blood Cancer J. 2024;14(1):206. 10.1038/s41408-024-01191-8 39592597 PMC11599389

[2] Moreau P , Garfall AL , van de Donk NWCJ , et al. Teclistamab in relapsed or refractory multiple myeloma. N Engl J Med. 2022;387(6):495‐505. doi:10.1056/NEJMoa2203478 35661166 10.1056/NEJMoa2203478 PMC10587778

[3] San‐Miguel J , Dhakal B , Yong K , et al. Cilta‐cel or standard care in lenalidomide‐refractory multiple myeloma. N Engl J Med. 2023;389(4):335‐347. doi:10.1056/NEJMoa2303379 37272512 10.1056/NEJMoa2303379

[4] Martin T , Usmani SZ , Berdeja JG , et al. Ciltacabtagene autoleucel, an anti‐B‐cell maturation antigen chimeric antigen receptor T‐cell therapy, for relapsed/refractory multiple myeloma: CARTITUDE‐1 2‐year follow‐up. J Clin Oncol. 2023;41(6):1265‐1274. doi:10.1200/JCO.22.00842 35658469 10.1200/JCO.22.00842 PMC9937098

[5] Costa LJ , Bahlis NJ , Perrot A , et al. Teclistamab plus daratumumab in relapsed or refractory multiple myeloma. N Engl J Med. 2026;394(8):739‐752. 10.1056/NEJMOA2514663 41363801 PMC13218738

[6] Kish J , Liu R , Pfeffer D , Vennam S , Lussier C , Nayak P . Real‐world duration of hospitalization for CAR‐T treatment: U.S. patient experience in multiple hematologic malignancies. J Clin Oncol. 2023;41(16_suppl):e18896. 10.1200/JCO.2023.41.16_SUPPL.E18896

[7] Hansen D , Liu YH , Ranjan S , et al. CT‐452 outpatient vs inpatient administration of chimeric antigen receptor (CAR) T‐cell therapy in cancer patients: systematic literature review. Clin Lymphoma Myeloma Leuk. 2023;23:S531. 10.1016/S2152-2650(23)01517-3

[8] Patel AR , Hasegawa K , Pandya S , et al. Health care resource utilization and costs of patients with diffuse large B‐cell lymphoma receiving chimeric antigen receptor T‐cell therapies across different settings of care: a real‐world data analysis. J Manag Care Spec Pharm. 2025;31(11):1110‐1122. doi:10.18553/jmcp.2025.31.11.1110 41171063 10.18553/jmcp.2025.31.11.1110 PMC12577723

[9] Bansal R , Paludo J , Hathcock MA , et al. Outpatient practice pattern for recently approved CAR‐T in patients with lymphoma and multiple myeloma. Blood. 2022;140(suppl 1):2399‐2401. 10.1182/BLOOD-2022-167187

[10] Bansal R , Paludo J , Corraes A , et al. Outpatient management of CAR‐T and teclistamab for patients with lymphoma and multiple myeloma. Blood. 2023;142(suppl 1):253. 10.1182/BLOOD-2023-187186

[11] Klastersky J , Paesmans M , Rubenstein EB , et al. The multinational association for supportive care in cancer risk index: a multinational scoring system for identifying low‐risk febrile neutropenic cancer patients. J Clin Oncol. 2000;18(16):3038‐3051. 10.1200/JCO.2000.18.16.3038 10944139

[12] Sidana S , Reid B , Dima D , et al. Enhancing the safety of ciltacabtagene autoleucel in relapsed multiple myeloma (MM): identification of potentially modifiable risk‐factors associated with delayed neurotoxicity and non‐relapse mortality. Blood. 2025;146(suppl 1):1034. 10.1182/BLOOD-2025-1034 40875552

[13] Woo JS , Nguyen K , Liu L , Krishnan A , Siddiqi T , Borogovac A . Mobilizing CARs: benefits, drawbacks, and directions for outpatient CAR T‐cell therapy. Sem Hematol. 2024;61(5):273‐283. 10.1053/J.SEMINHEMATOL.2024.08.003 39327109

[14] Munshi N , Martin T , Usmani SZ , et al. S202: CARTITUDE‐1 final results: phase 1B/2 study of ciltacabtagene autoleucel in heavily pretreated patients with relapsed/refractory multiple myeloma. HemaSphere. 2023;7(S3):e6102468. 10.1097/01.HS9.0000967720.61024.68

[15] Sidana S , Patel KK , Peres LC , et al. Safety and efficacy of standard‐of‐care ciltacabtagene autoleucel for relapsed/refractory multiple myeloma. Blood. 2025;145(1):85‐97. 10.1182/BLOOD.2024025945 39365257 PMC11952008

[16] Hansen DK , Liu YH , Ranjan S , et al. The impact of outpatient versus inpatient administration of CAR‐T therapies on clinical, economic, and humanistic outcomes in patients with hematological cancer: a systematic literature review. Cancers. 2023;15(24):5746. doi:10.3390/cancers15245746 38136292 10.3390/cancers15245746 PMC10741664

